# Template Imprinting Versus Porogen Imprinting of Small Molecules: A Review of Molecularly Imprinted Polymers in Gas Sensing

**DOI:** 10.3390/ijms23179642

**Published:** 2022-08-25

**Authors:** Todd Cowen, Michael Cheffena

**Affiliations:** Department of Manufacturing and Civil Engineering, Norwegian University of Science and Technology, 2815 Gjøvik, Norway

**Keywords:** molecularly imprinted polymers, gas sensors, volatile organic compounds, carbon capture, vapour, solvent, pollution monitoring, nanotechnology, polymer synthesis, plastic antibodies

## Abstract

The selective sensing of gaseous target molecules is a challenge to analytical chemistry. Selectivity may be achieved in liquids by several different methods, but many of these are not suitable for gas-phase analysis. In this review, we will focus on molecular imprinting and its application in selective binding of volatile organic compounds and atmospheric pollutants in the gas phase. The vast majority of indexed publications describing molecularly imprinted polymers for gas sensors and vapour monitors have been analysed and categorised. Specific attention was then given to sensitivity, selectivity, and the challenges of imprinting these small volatile compounds. A distinction was made between porogen (solvent) imprinting and template imprinting for the discussion of different synthetic techniques, and the suitability of each to different applications. We conclude that porogen imprinting, synthesis in an excess of template, has great potential in gas capture technology and possibly in tandem with more typical template imprinting, but that the latter generally remains preferable for selective and sensitive detection of gaseous molecules. More generally, it is concluded that gas-phase applications of MIPs are an established science, capable of great selectivity and parts-per-trillion sensitivity. Improvements in the fields are likely to emerge by deviating from standards developed for MIP in liquids, but original methodologies generating exceptional results are already present in the literature.

## 1. Introduction

Gas sensor elements based on molecularly imprinted polymers (MIPs) have several advantages over alternative technologies in their durability and selectivity [1,2,3,4] and have previously demonstrated application in food analysis [5,6,7,8], explosives detection [9,10,11], medical diagnosis [12,13,14], and pollution monitoring [15,16,17]. Detection limits of MIP sensors may be in the ppt range [18,19,20], interferent binding can be negligible [21,22,23], and the range of possible target molecules is extremely broad.

There are several excellent reviews of the use MIPs in gas sensors, but they principally focus on a specific technology or technological application. Reviews of specific techniques, for example, include the use of MIPs in gravimetric sensors and solid phase microextraction [3,13], while reviews of applications include targeting of a specific molecule [9,17], or use in a specific industrial setting [6,15]. The specificity of these reviews require discrimination in the selection of relevant articles to discuss. There is therefore a necessity for a broad review of all gas phase applications of MIPs, including all indexed publications on the field. It is also notable that these reviews rarely include any discussion of the potential for porogen imprinting in the resultant gas sensor [2]. As this is an effect which disproportionately affects gas-phase applications, this was chosen as a framework for an extensive review of MIPs in gas sensing.

‘Porogen imprinting’ [24] (or ‘solvent imprinting’ [25]) is an infrequently used term which refers to molecular recognition of polymers for their porogen of synthesis. This will be contrasted with ‘template imprinting’ (or ‘authentic imprinting’ [26]) to describe cases where the template is added in low concentrations and contributes minimally to the solvation of the monomer/polymer. Template imprinting is therefore the conventional technique and involves imprinting by polymerisation around the template. Porogen imprinting meanwhile is imprinting in what would by convention be considered an excess of template. This will be discussed in greater detail in the following sections.

Porogen imprinting in gas sensors is well represented in the literature. A review was performed to gather and evaluate the vast majority of publications describing MIP-based gas sensors. The results are presented in Table 1. Each article is represented by the gas phase target, grouped by the number of atoms in the molecule. This gives an approximation of volume and correlates with structural complexity, which are more relevant to imprinting than molecular mass or volatility. Each example is then categorized as template imprinting or porogen imprinting. Methods in which the imprinting molecule is explicitly described as the solvent, or where it exists in a greater quantity than all monomer or polymer reagents, will be considered porogen imprinting. Additional comment will then be made on the polymer structure, grouped broadly into nanoparticles (including some examples of microparticles produced by similar methods), films (including coatings and electropolymerised polymers) or monoliths (typically ground monoliths). These comments on polymer structure are intended to provide an indication of the synthetic techniques applied for each size classification.

The table naturally consists predominantly of volatile organic compounds (VOCs) with a smaller number of common gases. The ratio of nanoparticle to monolith protocols is consistent through the different size categories, but the prevalence of films increases with molecular complexity. This is partially due to the preference for film synthesis in template imprinting, porogen imprinting more commonly employing nanoparticle or bulk (monolith) synthesis. MIPs for small (≤10 atoms) molecules were produced by porogen imprinting in 46% of cases, but this proportion decreases with larger targets.

The distinction between porogen imprinting and template imprinting used here may be important. Template (conventional) imprinting is a process resulting in a specific and discreet binding site with affinity significantly greater than that observed by non-selective binding at a non-imprinted site on the same material. Porogen imprinting is the retention of affinity for a specific solvent by a material, on the surface of that material and within pores [20]. The emphasis on monomer-template ratio here is a product of standard imprinting theory, in that affinity and selectivity are presumed to derive from the interaction of multiple polymer functionalities in the binding site [179,180]. Multiple studies suggest the optimal functional monomer-template ratio is 4:1 for most applications [164,179,181]. This does not include the crosslinker, which is often the major component of the polymer. If the template concentration exceeds that of the monomers, it is inherently excessive, and the proportion of polymer available for each template is diminished.

Both template and porogen imprinting however derive affinity for a specific molecule from the material being synthesised in its presence. They are therefore both forms of molecular imprinting, and both present possibilities in various areas in which MIPs are applied. This review will focus primarily on imprinted polymers for binding gas phase targets, and secondarily on comparing template and porogen imprinting. A brief overview will first be given contrasting the preparation of MIPs for gas phase applications and more common techniques with solutions. This will be followed by sections on porogen imprinting and template imprinting, each containing examples of each technique grouped into relevant subsections.

## 2. Molecular Imprinting of Volatile Molecules

Molecular imprinting, both in synthesis and application, depends on binding to the template/target. Affinity and selectivity are both dependent partially on the number of points of interaction with the template/target. Synthesis of MIPs with significant affinity and selectivity for small molecules is therefore inherently more difficult than preparation for conventional targets. High volatility precludes strong intermolecular interactions. This may explain the bias towards larger targets observable in Table 1, despite the generally reduced socio-economic benefits of this. Even in the examples of larger volatile targets, the molecule typically has few functionalities with which monomer or polymer functionalities can bind. Conventional functional monomers (e.g., methacrylic acid) may preferentially dimerize over binding to many common VOCs (pyridine, benzene, etc.) [182] Meanwhile, MIPs in gas-phase analysis typically give greater non-specific binding of interferent compounds that equivalent MIPs in liquid [170].

Detailed theoretical studies of gas adsorption by imprinted polymers are also rare [98]. It is difficult to determine general models of MIP gas sensors due to the chemical and macroscopic variation between polymers, in addition to dependence on targets and environmental conditions [183]. It has not been determined how dependent MIPs may be on solvent in the binding site, which will not be present in the gas phase [184]. It has been observed that binding sites may be lost with time in the gas phase, though other MIP sensors appear to be resilient to this [131]. It is even possible that polymers synthesised in the presence of a very small template forms no bonds with template, in the same way that water is not greatly disrupted by dissolved small alkanes [185].

There are also mechanistic considerations relating to polymer synthesis. Phase separation during polymerisation can be suppressed by decreasing the relative crosslinker concentration, decreasing the concentration of all components, or increasing the solubility of the polymer [105,186,187]. Preventing phase separation results in a gel-type polymer which is still essentially soluble in the porogen. Gel-type polymers dry to form amorphous glassy materials but swell significantly in specific solvents [187]. Encouraging phase separation meanwhile results in macroporous polymers, with a greater relative surface area. With lower concentrations, in nanoparticle synthesis, earlier phase separation is associated with smaller particles [40,188]. Phase separation is accelerated by the presence of nucleation sites, which include the template with conventional ratios [105,180,188]. Seed polymers can be used in a similar manner to accelerate phase separation, and can results in greater porosity and target binding [189].

Ultimate polymer properties therefore depend on a complex combination of variables, dominated by the interactions and concentrations of monomer, template and solvent. This is generally simplified in porogen imprinting, as the template and solvent are typically the same. However, whether porogen imprinting is desirable, being more suited to certain purposes than conventional template imprinting, has rarely been discussed. The distinction between porogen and template imprinting is principally one of relative concentration, as both template and porogen are fundamentally solvents in polymerisation [105]. It is therefore reasonable to assume that porogen imprinting would result in more effective MIPs, due to the greater opportunity for imprinting. This is particularly relevant in gas phase sensing, where all target binding is likely to be weak.

**Figure 1:**
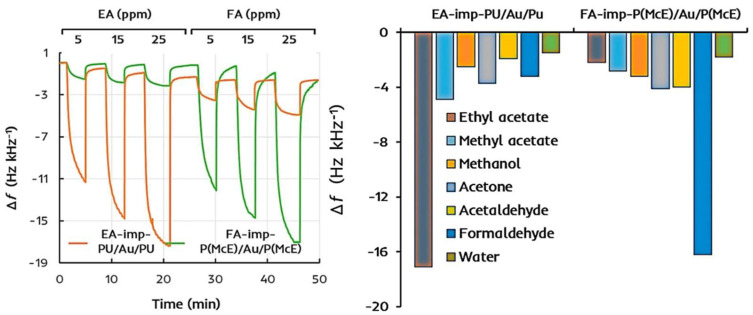
Ethyl acetate (EA) and formaldehyde (FA) imprinted polymers produced by porogen and template imprinting, respectively. The sensors were produced by coating a QCM device with imprinted polymer (polyurethane, PU, or methacrylic acid-co-ethylene glycol dimethacrylate, McE), followed by coating with gold nanoparticles, and finally with a second layer of MIP. The change of resonance frequency (Δ*f*) was then monitored in the presence of different gaseous compounds in dry air. Studies with interferents (right) were performed with 25 ppm analyte, or 50% relative humidity in the case of water. Reproduced with permission [44].

A rare example of both template and porogen imprinting being applied by the same protocol demonstrates the uncertainty. Afzal et al., prepared MIPs for ethyl acetate and formaldehyde, using ethyl acetate as both template and porogen in the former, and bubbling formaldehyde through THF in the latter [44]. The results show good selectivity and high sensitivity for each MIP, as shown in Figure 1. In this example however, the ethyl acetate MIP was produced by porogen imprinting, while the formaldehyde MIP was produced by a more conventional template imprinting approach. The precise synthetic mechanisms are therefore likely to be different, but the results are similar.

Whether porogen imprinting or template imprinting is preferable is therefore not immediately clear. For this reason, the following sections of this review are divided into examples of porogen and template imprinting. Specific attention is given to selectivity and sensitivity, as these are popular parameters for the evaluation of molecularly imprinted polymers. Conclusions are given at the end of each section and at the end of the article.

## 3. Porogen Imprinting

It has been well established that solvents can have a profound impact on the properties of polymers. For example, linear polymers annealed in chiral solvents can result in chiral nanoparticles [190]. In MIPs, the interactions between the template and the monomer/polymer are dominated by the characteristics of the porogen [24,186]. We have previously discussed how the mesoscopic structure and synthetic mechanism are determined by solvent [180,188]. and others have demonstrated that the porogen can greatly affect the total binding capacity, imprinting factor and the selectivity [191].

Preparation of a polymer in the presence of a molecule can induce a binding site for that molecule in the polymer. Under optimal conditions, this is simply a result of achieving the most energetically favourable intermolecular interactions between the polymer and the molecule. This process may occur with any molecules present, and (as will be shown) does not necessarily even require the polymer to undergo a chemical reaction. Porogens (solvents) are usually chosen for their affinity (dissolving ability) for the monomer, and affinity is retained in gels and similar materials.

**Figure 2:**
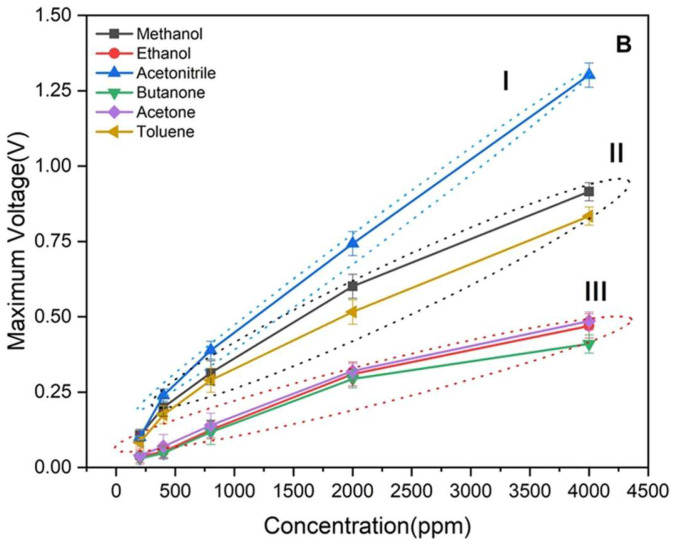
Janfaza et al., produced a MIP via conventional template imprinting for acetone, but the polymer shows porogen imprinting for acetonitrile. MIPs were synthesized by the polymerization of ethylene glycol dimethacrylate and methacrylic acid with acetone (3:1:1) in dry acetonitrile. The resulting polymer was ground to powder, dispersed in acetonitrile and drop-casted onto an electroactive microfluidic sensor, permitting monitoring resistance variation. I, II and III refer to different clusters of responses determined from this result. Reproduced with permission [41].

This affinity for solvents of synthesis (porogen imprinting), can be easily demonstrated. Figure 2 shows the response of a MIP nanoparticle-coated chemiresistive sensor for acetone [41]. The template acetone and functional monomer methacrylic acid were mixed in a 1:1 ratio in acetonitrile, before addition of crosslinker and initiator, followed by polymerisation. The resulting MIP however showed no selectivity for acetone over similar alcohols and ketones, but a much greater response to acetonitrile. The sensor therefore exhibits porogen imprinting behaviour, without the researchers attempting to achieve this result. This effect however can be exploited and optimized for the development of sensors using the target analyte as a porogen.

Porogen imprinting was used in some of the earliest examples of MIPs for gas phase analysis, notably in Dickert’s quartz crystal microbalance (QCM) and surface acoustic wave (SAW) gravimetric sensors for various VOCs [62,63,136,192]. Amongst these were examples of selectivity in the presence of physically similar compounds (ethyl acetate and ethanol) and sensitively to 0.1 ppm variations in vapour concentration [62]. The group also produced an optical vapour sensing device which changes colour in response to the imprinted solvent [63]. In the earliest of these publications, which is amongst the earliest examples of imprinting for gas phase targets, Dickert et al., made sensors with selectivity for a specific xylene isomers by the imprinting of that isomer [136]. In each of these cases the target molecule is also the polymerisation solvent, meaning porogen imprinting can be found in the earliest MIP gas sensors. Reviews of MIP gas sensors from this time describe the imprinted molecule as the porogen [193].

**Figure 3:**
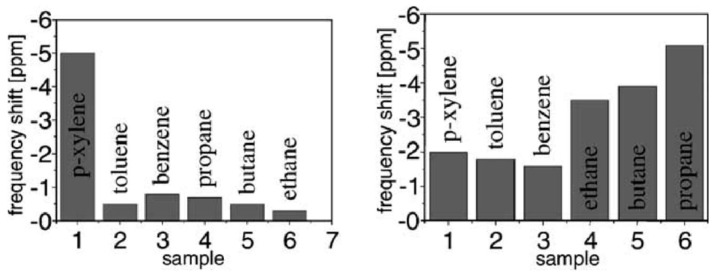
*p*-Xylene imprinted SAW sensor response to VOCs (**left**) and response from a non-imprinted equivalent (**right**). Both polymers were prepared from polyurethane to give a layer of approximately 40 nm, but only the imprinted polymer used *p*-xylene as solvent. Reproduced with permission [137].

**Figure 4:**
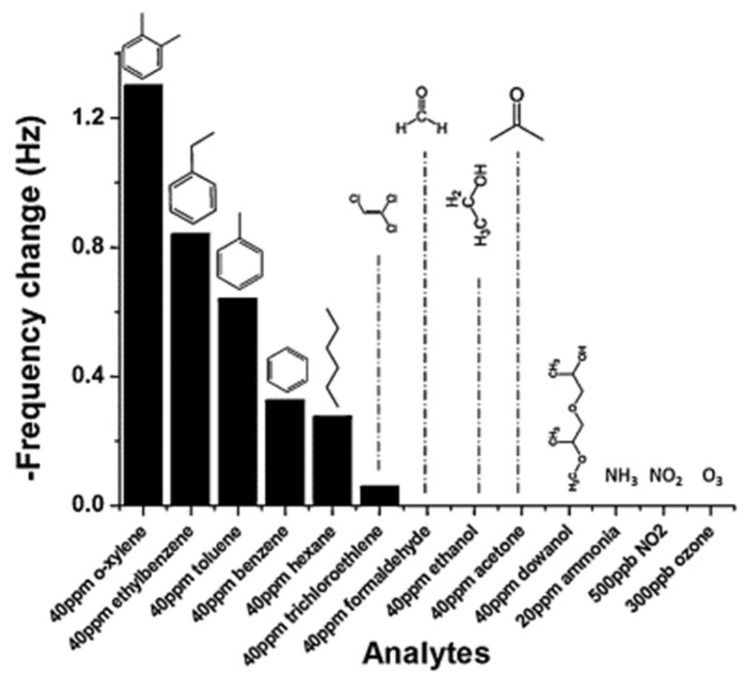
Results of Deng’s selectivity studies in analysis of a porogen imprinted polymer for *o*-xylene. MIPs were produced using divinylbenzene as functional monomer and crosslinker, and *o*-xylene was used as template and solvent. The resulting monolith was ground, dispersed in *o*-xylene and cast onto a quartz crystal to give a gravimetric sensor. Reproduced with permission [131].

QCM and SAW based gas sensors have been prepared to great effect with porogen imprinted recognition sites. Wen et al., developed a SAW based sensor using sarin acid (template) and *o*-phenylenediamine (monomer) in a 3:1 ratio [141]. A film of 10 nm thickness was produced following electropolymerisation by cyclic voltammetry and template extraction with deionized water. The molecular imprinting process in this research was therefore relatively simple, but the sensor still gave an LOD of 0.1 ppm to the target dimethyl methylphosphonate. Other examples of early SAW-based sensors demonstrate the selectivity that can be achieved with porogen imprinting, as in the work Bender et al., with sensors for toluene and *p*-xylene [137]. The results of selectivity tests of the *p*-xylene sensor are given in Figure 3, demonstrating the specific binding of *p*-xylene over similar aromatic VOCs. Selective sensors based on porogen imprinting of *o*-xylene (Deng et al., Figure 4 [131]) and toluene (Alizadeh and Rezaloo [123]) show this selectivity is a result of imprinting, and not simply an anomaly arising from the target.

**Figure 5:**
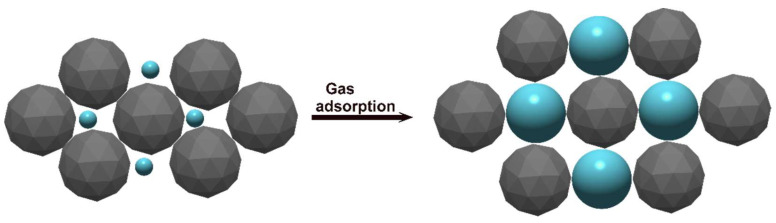
Scheme of a MIP nanoparticle-based gas sensor, in which target-induced swelling of molecularly imprinted polymer particles (blue) increases the electrical resistance by separating conductive materials (grey). This principle is the basis of sensors using conductive materials such as carbon nanotubes, graphene, or metal particles, and the polymer may be present as nanoparticles, microparticles, or continuous film.

In the latter example of Alizadeh and Rezaloo, the dynamic range of this sensor was 3.8–46.4 ppm and the limit of detection was 0.8 ppm. It also involved an electrochemical method, which may be preferable to the mass sensing methods previously described. Porogen imprinting in electrochemical sensors typically relies on polymer swelling in the presence of the target molecule, often aided by the addition of conductive nanomaterials. Early studies suggested that dependence on this mechanism may cause problems with sensor consistency due to polymer deformation on repeated use [194]. This is apparently a concern primarily with polymer films, where porogen imprinting is likely to give a uniform featureless surface [189]. Such materials are more susceptible to stresses of swelling and provide a relatively low surface area. More common therefore is the use of microscopic porogen imprinted polymer spheres with the conductive material. An illustration is given in Figure 5 [82]. Cheap conductive materials such as carbon black may be used for this purpose. More sophisticated methods involving very small nanoparticles (10 nm) and carbon nanotubes may give sub-ppm sensitivity and high selectivity [57].

### 3.1. Selectivity of MIPs Produced by Porogen Imprinting

Studies of porogen imprinting with *o*-xylene and *p*-xylene demonstrated selectivity of the polymer for its specific isomer [24]. However, a greater affinity for other aromatic compounds of the equivalent structure is also observed. For example, *o*-dichloromethane bound preferentially to *o*-xylene imprinted polymers relative to *p*-chloromethane. However, this effect is greatly reduced with difluorobenzene and dinitrobenzene. This suggests that porogen imprinting is dependent primarily on the specific shape and volume of the porogen, and not simply on π-π interactions or hydrogen bonding [24].

Matsuguchi and Uno demonstrated that increasing the solvent-monomer ratio to 5:1 from 1:1 causes an increase in solvent/template adsorption in rebinding studies [122]. This analysis also showed that a simple methacrylic acid and divinylbenzene polymer (5:1, ground monolith) synthesized in toluene will preferentially rebind toluene over *p*-xylene, and that synthesizing the polymer in *p*-xylene would double that affinity for *p*-xylene.

**Figure 6:**
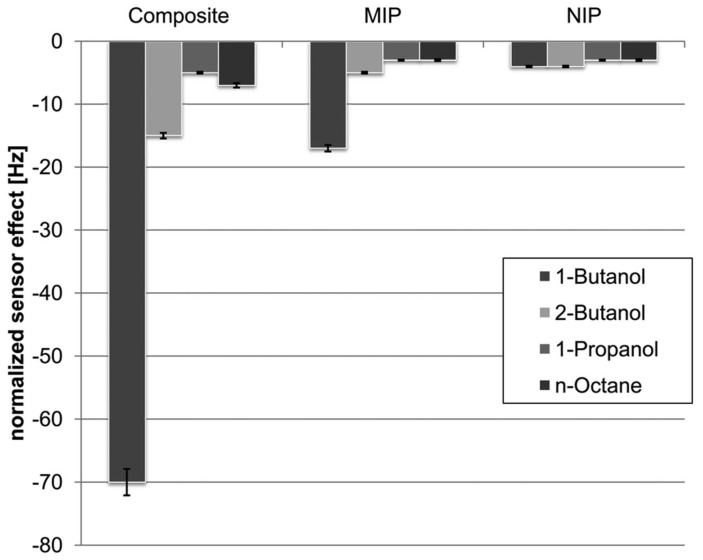
Results of selectivity studies (400 ppm) with a 1–butanol porogen imprinted polymer–Ag_2_S nanoparticle composites. Polyurethane MIPs were synthesized in 1–butanol with Ag_2_S particles (50 nm), and spin-coated onto a quartz crystal microbalance. Comparison was made between the MIP composite produced in this manner, MIPs produced without the Ag_2_S nanoparticles, and a non-imprinted equivalent prepared using tetrahydrofuran. Reproduced with permission [138].

Studies of these simple aromatics and their sensitivities are common in the literature [121,125]. However, studies with other targets may be more dramatic. MIP-Ag_2_S nanoparticle composites were produced by Mustafa and Lieberzeit for the selective detection of 1-butanol by a porogen imprinting technique [138]. A combination of monomers were diluted in a large excess of 1-butanol with Ag_2_S nanoparticles of approximately 50 nm diameter. Equivalent non-imprinted (‘NIP’) mixtures were prepared with THF, as were mixtures without nanoparticles (‘MIP’). Applying each composite as a QCM sensor film, the resulting selectivity is demonstrated by the results in Figure 6. From this it can be observed that the MIP (porogen imprinted polymer) has some selectivity for the target 1-butanol over structural similar compounds, including 2-butanol and 1-propanol. More obvious however is the increase in this response in the presence of the Ag_2_S nanoparticles, which exaggerates the selectivity by increasing the sensitivity of the apparatus to a 2 ppm limit of detection. This is amongst the best examples of the selectivity that can be achieved with porogen imprinting.

**Figure 7:**
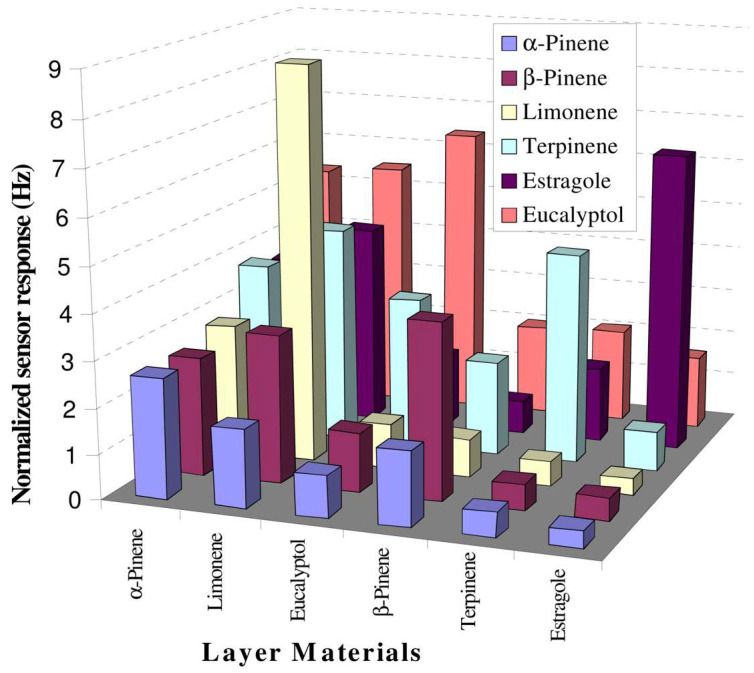
Sensitivity and selectivity of identically produced porogen imprinted polymers with different templates. Some MIPs (layer materials) show a strong selectivity and response to their imprinting molecule, while others do not. QCM–MIP sensors were produced using an experimentally determined optimal composition of 1:1.5:10 functional monomer (styrene) to crosslinker (divinylbenzene) to template ratio. Reproduced with permission [140].

However, selectivity in porogen imprinting is generally more variable than template imprinting. Iqbal et al., produced MIP-QCM sensors for several terpenes, and presented the response observed for each MIP to each terpene template [140]. The results in Figure 7 (response for each terpene at 50 ppm) demonstrate the variability in sensitivity and, more strikingly, selectivity that may result from identical methods and similar templates. For example, the estragole imprinted film (far right) shows high selectivity for estragole, giving a strong response to only this terpene. The film produced by α-terpene imprinting however (far left) shows no selectivity for its target, apparently giving a greater response to several of the interferents. These results may partially be explained by a phenomenon observed with higher ratios of larger targets, in which the terpene may impede polymerisation [159]. The selectivity observed therefore may not be representative of porogen imprinting generally due to the use of large targets.

### 3.2. Sensitivity of MIPs Produced by Porogen Imprinting

Porogen imprinted polymers have also been developed with sensitivities down to individual ppb [135]. Examples of this include a porogen imprinted polymer amongst the most sensitive of MIPs discoverable in the literature. Adams et al., produced a MIP by the electropolymerisation of phenol (5 mM) with methyl salicylate (template, 28 mM) onto a single layer of graphene [20]. The resulting sensor gave a response proportional to methyl salicylate concentration in the sub-ppb range, down to a limit of detection of approximately 10 ppt (parts-per-trillion). The researchers had previously used a similar technique to produce sensors for butylated hydroxytoluene [165]. One of these sensors similarly gave a response proportional to ppt concentrations of the target with an LOD of 20 ppt.

High sensitivity was also achieved by Shim et al., in producing MIPs for toluene and phenol. This technique involved dissolving linear polymers in an aqueous-toluene emulsion and deposition to give a QCM sensor [124]. The limit of detection was reportedly 33 ppb under standard conditions, and 6.6 ppb in a controlled environment. Linear polymers of this nature can be effective in retaining a memory for their last solvent of dissolution [56]. This may be more reliable for porogen imprinting; a polymer dissolved in a dilute solution of a template does not necessarily result in selectivity or affinity for that template, and linear polymer additives may inhibit binding [78,189,195]. In the case of porogen imprinting, the polymer is already fully formed, but adopts an arrangement most suitable to maximize favourable interaction with the solvent. On solvent evaporation, this configuration is partially retained, leaving sites for the target to rebind and resolvate the polymer [194].

### 3.3. Small Molecule Porogen Imprinting

Selectivity for smaller molecules is more difficult to achieve with imprinting than it is with more complex molecules as the imprinting effect is generally proportional to the molar volume [196] The difficulties of imprinted small molecules, and the consequent adoption of porogen imprinting however, have been discussed for some time [122] This may be best demonstrated by some of the work by Rong and associated. The first of these demonstrated an effective acetone sensor by polymerising in a mixture of acetone and water [69]. The sensor shows high selectivity and sensitivity, as demonstrated by the response to 5 ppm of various gases. Under these conditions, the optimal sensor shows a response approximately 10 times greater to acetone than formaldehyde, and greater still compared to methanol and other VOCs.

**Figure 8:**
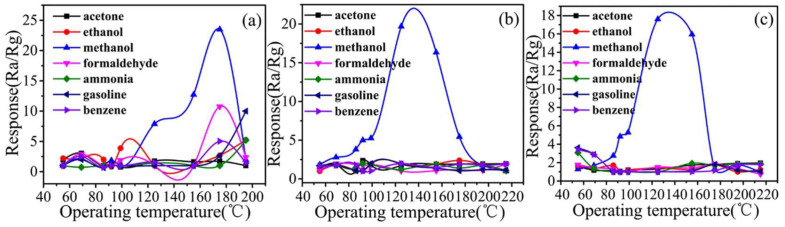
Methanol sensors produced by Rong et al., on exposure to different VOCs at 5 ppm. The three sensors were produced using different support fibres: paper (**a**), silk (**b**), or cotton (**c**). Methacrylic acid and an Ag-LaFeO_3_ sol were reacted in methanol to give a gel used to coat the different supports. Each material was then ground and printed onto an alumina electrode, and the response calculated as the ratio of the electrical resistance in the specified gas (Rg) and in air (Ra). Reproduced with permission [22].

The same researchers applied a similar method in the preparation of a methanol sensor, using a various support fibres (paper, silk, or cotton) coated with MIPs prepared from Ag-LaFeO_3_ and methacrylic acid [22]. The selectivity for methanol was high regardless of the fibre (Figure 8), showing a strong response to methanol at 5 ppm. The effect of the possible interferent is small to negligible at the same concentration, even in the case of structurally similar ethanol and formaldehyde. The sensor gave a linear response to methanol in the range 1-5 ppm, and a sub-ppm LOD. The researchers obtained similar results applying the same methodology to produce methanol imprinted microparticles [66].

### 3.4. Conclusions from Porogen Imprinting

Evaluations of porogen imprinting are limited by the lack of available and consistent data. It has been stated, and some of the previous examples support this, that the porogen imprinting effect is greater when the polymer has a low solubility in the solvent [25]. Meanwhile both the affinity and surface area should be proportional to the solubility [26]. Comparison with conventional template imprinting is therefore difficult, due to the degree of parameter optimization in standard MIP synthesis [197]. There is still a great requirement for optimal porogen imprinting conditions to be established.

It will be shown that improvements in selectivity and sensitivity are likely to be achieved with template imprinting. However, as is shown in Table 1, porogen imprinting may be more appropriate than template imprinting for the preparation of sensors for common solvent vapour. They may also be more useful generally in capturing certain atmospheric pollutants, due to the greater binding capacity inherent in porogen imprinting. It may also be possible for this technique to be employed alongside template imprinting to give the MIP affinity for two molecules simultaneously. Examples of mechanism like this already exist, such as the carbon dioxide responsive switchable polymers selective for proteins, prepared by synthesis in the presence of excessive CO_2_ [198,199,200]. For less ambitious projects involving gas sensing however, template imprinting is likely the better option where possible.

## 4. Template Imprinting

While the details are lacking in porogen imprinting, the basic mechanisms of sensing are relatively simple. If the polymer is optimized for affinity to a specific solvent, re-exposure to that solvent likely gives adsorption. The adsorption is proportional to the concentration, and to the mass and volume of the adsorbed material. The sensors mechanism then exploits this change in mass or volume. With conventional imprinting in which the template concentration in the pre-polymerisation solution is lower, the number of template induced binding sites is reduced. This generally makes the previous explanation unsuitable, especially for small gaseous targets.

The response to binding in conventional template imprinting is a more complex effect resulting from the specific affinity of the binding site for the target [201]. In electrochemical sensors, the result of target binding is a strong disruption in local electron density which propagates through the polymer. In mass-based analysis (QCM, SAW) the equilibrium is shifted as far as possible to target-polymer association. In all cases therefore, template imprinting depends on high affinity binding to the target, in a manner that is less necessary in porogen imprinting. The relatively higher ratio of monomer to template ensures maximal interaction. The high monomer-template ratio thus creates the requirement and the means of achieving high target affinity.

**Figure 9:**
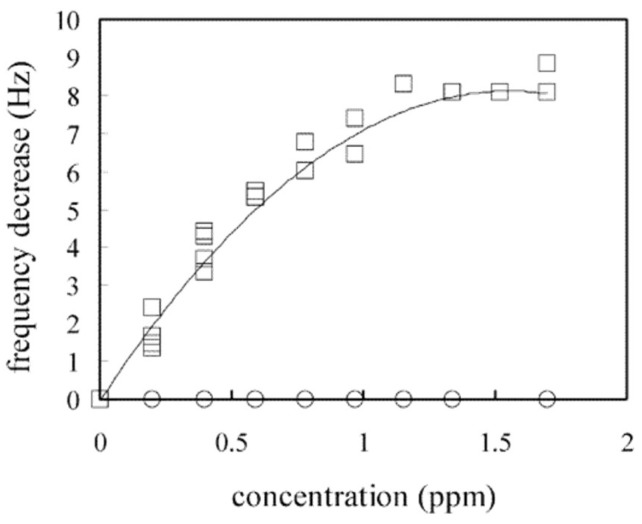
QCM response to menthol imprinted polymer films (squares) and non-imprinted controls produced with the same monomers (circles). The results shown were produced using a template, monomer (methacrylic acid), crosslinker (ethylene glycol dimethacrylate), solvent (chloroform) ratio of 1:4:20:50. Following polymerization the MIP was cast on a quartz crystal and measured gravimetrically. Reproduced with permission [172].

As with porogen imprinting, conventional imprinting methods have been used in MIP gas sensor production since the late 1990s [171]. In an early example, Percival et al., prepared an L-menthol imprinted polymer for a QCM-based sensor [172]. The MIP was prepared using a 1:4 ratio of menthol to methacrylic acid, with studies of various crosslinkers. The highest crosslinker ratio was found to be most successful (3:20 functional monomer to crosslinker), and a response was observed proportional to L-menthol concentrations to a limit of 200 ppb. Good selectivity was also observed for the specific target when contrasted with similar terpenes and the menthol stereoisomer. The imprinting factor was particularly high, with the non-imprinted control showing negligible binding (Figure 9).

**Figure 10:**
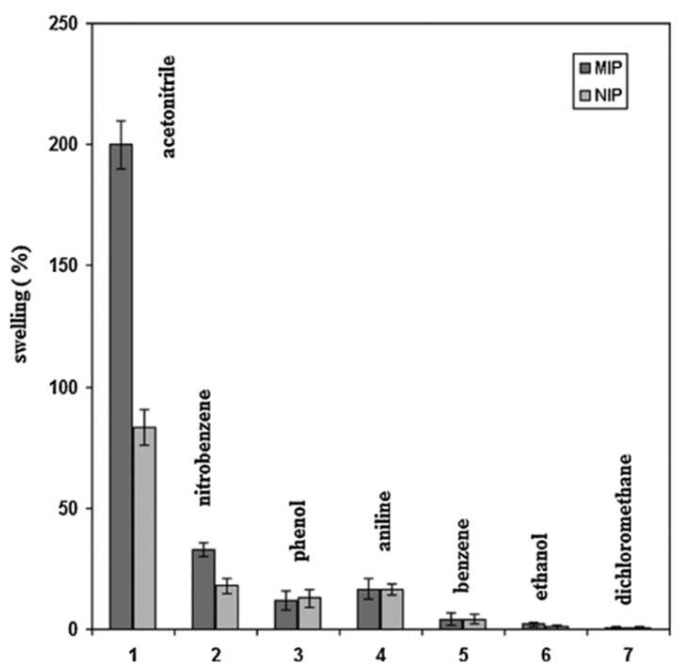
Swelling intensity of imprinted and non-imprinted polymers in the presence of different VOCs. MIPs are imprinted for nitrobenzene, both MIPs and NIPs are prepared using acetonitrile as porogen Reproduced with permission [203].

Such examples of template imprinting became relatively common in time. What is generally ignored however is the possible effect of porogen imprinting which may occur during the MIP synthesis. In solution, non-specific binding may be independent of the solvent used in the polymerization [202]. In the gas phase however, greater attention may be required to avoid inadvertent porogen imprinting. An example can be found in the MIP for nitrobenzene prepared in acetonitrile by Alizadeh et al. [203]. The resulting sensor gave an impressive LOD of 200 ppb, and a linear response to the target in the concentration range 0.5–60 ppm. The authors also however presented the results of polymer swelling in different solvents, showing a dramatic effect in the synthesis solvent (acetonitrile, Figure 10). The additional swelling of the MIP compared to the non-imprinted polymer (NIP) is explained as being a result of the greater porosity of the former, an effect that has been noted by other researchers [76,158]. Increasing porosity may increase both sensitivity and selectivity regardless [16]. Notable too is that nitrobenzene, the template, showed the second greatest swelling response after acetonitrile. The authors had previously noted the swelling of polymers in the solvent of their synthesis, and the additional swelling of imprinted nanoparticles (and not their non-imprinted equivalents) in the presence of the template [204].

From this it is clear that the porogen can have a great effect on the final MIP, and sufficient measures should be introduced to prevent interference from rebinding the porogen. This may not be difficult, as porogen selectivity is not inevitable [191]. Secondly, however, the template has a great effect on the properties of the final polymer. The imprinting process results in a material with much greater swelling in both the presence of the template, and in the presence of the porogen. Even in relatively low concentrations therefore, the template has a dramatic effect on the physico-chemical properties of the polymer. As in the example of porogen imprinting in the previous section, MIP nanoparticle target-induced swelling can be correlating with electrical resistance for sensor fabrication [40,156]. Template imprinting however results in polymer properties distinct from both standard polymer preparation and porogen imprinting, disproportionate to the scale of interaction. This makes them excellent as sensitive and selective gas sensors.

### 4.1. Selectivity of MIPs Produced by Template Imprinting

**Figure 11:**
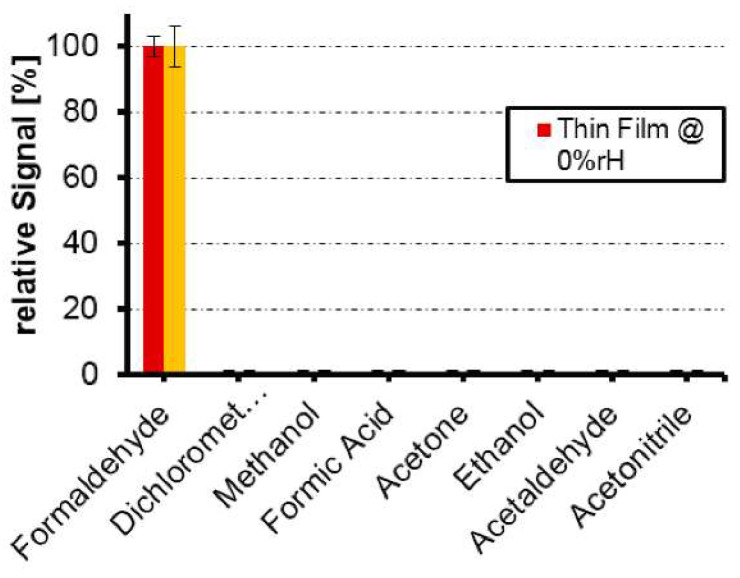
Formaldehyde sensor produced by Hussain et al., with several gases at 100 ppm. The graph is striking but somewhat superfluous as it simply shows formaldehyde at 100% and all other VOCs at 0%. MIP films (red) and nanoparticles (yellow) were prepared using an approximately 1:1:1:2 template-monomer-monomer-crosslinker ratio in methanol and dimethylformamide. Films were spin-coated directly onto the QCM electrode, while nanoparticles were first precipitated by addition of acetonitrile. Reproduced with permission [21].

One of the best demonstrations of selectivity in gas sensors is the widely reported study by Hussain et al., in their preparation of a formaldehyde MIP [21,205]. MIP films and nanoparticles were produced in an approximate 1:1:1:2 ratio of template, functional monomer 1 (styrene), functional monomer 2 (methacrylic acid) and EGDMA crosslinker, with a relative excess of solvent. The resulting QCM-based sensor displayed an LOD of 500 ppb, making it an excellent example of sensitivity. More impressive however is the selectivity, in which various interferent VOCs, including methanol and formic acid (the reduced and oxidized product of the template), could not be detected above the noise level of the oscillator, even at concentrations of 100 ppm (Figure 11). Notable too is that methanol was 40 vol% of the binary solvent system used in the preparation of this MIP (results are not given for the other solvent, dimethylformamide), showing that the polymer does not necessarily display affinity for the synthesis solvent.

**Figure 12:**
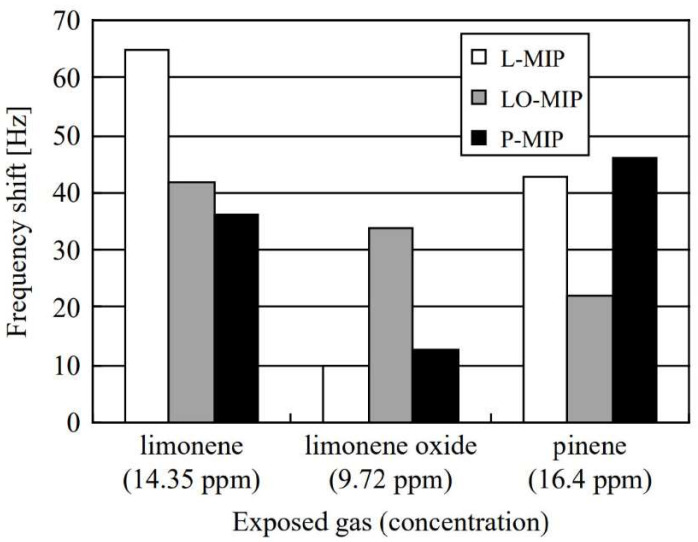
Response of QCM-MIP sensors for limonene, limonene oxide and pinene (L–MIP, LO–MIP and P–MIP) for each of the analytes. MIPs were prepared using a 1:4:20 ratio of template, methacrylic acid and ethylene glycol, which were then cast onto QCM surfaces following polymerization. Reproduced with permission [149].

Larger target compounds present a greater opportunity for selectivity tests, as a greater proportion of an analogue may be identical to the target. Kikuchi et al., provide an example of this in their preparation of MIPs for limonene, limonene oxide and α-pinene [149]. With a composition of simply methacrylic acid and ethylene glycol dimethacrylate with initiator and template, the group produced relatively sensitive instruments with good selectivity (Figure 12). The selectivity is particularly notable here due to the similarity of limonene and limonene oxide.

**Figure 13:**
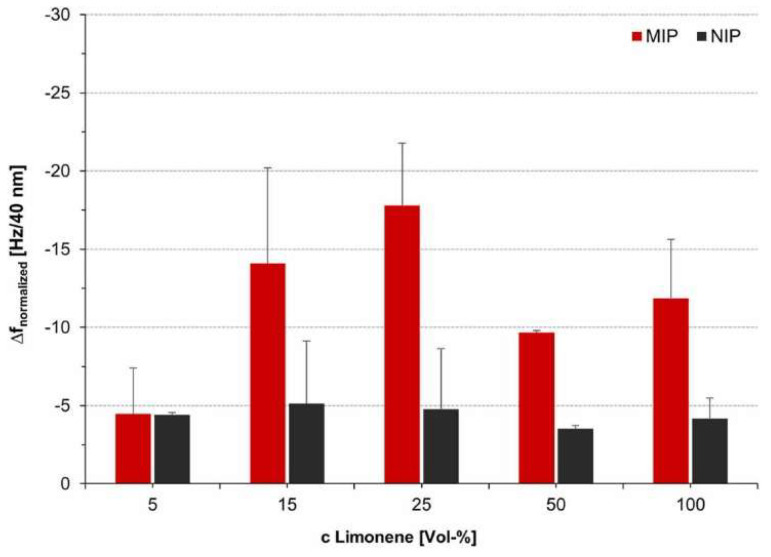
Effect of varying template concentration in the imprinting of limonene by Völkle et al., A total of 300 µL of styrene and 700 µL of divinylbenzene were combined in 1300 µL of limonene and toluene in differing ratios. The resulting MIPs were spin–coated onto QCM sensors and studied for their response to limonene at 250 ppm. Reproduced with permission [145].

Other researchers have similarly produced limonene selective MIPs using common reagents and ratios (1:4:20 template-monomer-crosslinker) [143,144]. More pertinent with regard to this review however is the analysis by Völkle et al., in their study of limonene imprinting with different template-solvent ratios [145]. Styrene and divinylbenzene monomers (300 μL and 700 μL, respectively) were dissolved in solutions of limonene and toluene (1300 μL), with the template being varied from 5% to 100% of the solution. The QCM response of each MIP sensor to 250 ppm limonene is given is Figure 13. The sensor response was found to increase with limonene concentration up to 25%, equivalent to approximately 2.0:2.6 molar ratio of limonene to styrene. The decline as the concentration is increased may be a result of limonene impeding polymerisation, and possibly being bound covalently to the polymer [145].

**Figure 14:**
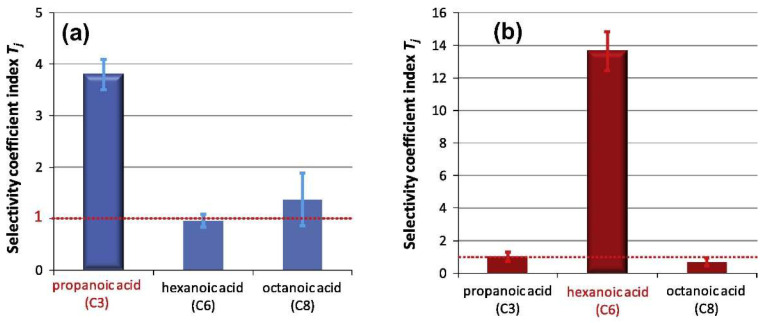
Binding of targets (red text) to their MIPs relative to non-imprinted polymers: (**a**) propanoic acid imprinted polymer binding to propanoic acid, hexanoic acid and octanoic acid relative to non-imprinted polymer, (**b**) hexanoic acid imprinted polymer binding to propanoic acid, hexanoic acid and octanoic acid relative to non-imprinted polymer. Reproduced with permission [23].

Applications of these protocols may be less suitable for smaller VOCs however, for which selectivity can become a problem [40]. This can be shown by the work of Imahashi and Hayashi, in their studies of MIPs for carboxylic acids of different lengths [23]. Figure 14 shows the selectivity coefficient (quantity of chemical adsorbed to MIP relative to NIP, imprinting factor) of propanoic acid and hexanoic acid imprinted polymers synthesised under otherwise identical conditions. While the selectivity of propanoic acid would be acceptable for many applications, it is extremely low compared to that of hexanoic acid, despite their functional similarity.

A sol-gel based sensor developed by Edmiston et al., for trinitrotoluene is amongst the best demonstrations of high sensitivity and selectivity [19]. This relatively early example of MIP-based gas sensing gave a limit of detection of 2.4 ppt. Selectivity tests resulted in a 50-fold greater response to trinitrotoluene than the structurally analogous 2,4-dinitrotolune, and 6 orders greater than the response to toluene. More recent studies have shown that discrimination of enantiomers is possible with gas phase targets using MIPs. [117]. However, the low sensitivity demonstrated by Edmiston may be more important for many applications, and this is generally where the improvements must arise for commercial viability.

### 4.2. Sensitivity of MIPs Produced by Template Imprinting

High sensitivity is a requirement for many applications, but the precise methods used to achieve this goal are often unconventional. There are studies suggesting that delayed addition of template to a polymerising mixture, in which there are functional oligomers not monomers, can greatly enhance the MIP selectivity [206]. This is perhaps similar to the examples of imprinting using pre-synthesized linear polymers dissolved in a dilute solution of template, followed by evaporation of solvent [38,109]. Highly sensitive examples resulting from this method include the use of linear polyaniline in the preparation of a formaldehyde gas sensor yielding high selectivity and a detection limit of 30 ppb [45]. The number of successful examples of this technique being used in gas sensor preparation, with templates from simple carboxylic acids to organophosphates, is possibly disproportionate to the field [37,82,85,86,114,116].

More analogues to delayed addition is the dissolution of template with linear polymer, followed by crosslinking. This was demonstrated by Koudehi et al., in their highly selective 2,4-dinitrotoluene sensor [11]. Polyvinyl alcohol (72,000 g/mol) was dissolved in water with glutaraldehyde and small quantities of template. Heating with HCl then gave a crosslinked polymer, which could be used to detect an explosive marker with high sensitivity, giving a The sensor reportedly gave a response proportional to analyte concentration in the range 10-1000 ppb. This represents a continuation of work by Koudehi et al., who previously demonstrated a sub-ppm explosives sensor with the same MIP reagents [107]. Niu et al., have similarly produced sensors for dinitrotoluene and trinitrotoluene using poly(fluorene-co-benzamide) grafted onto a cellulose film, followed by crosslinking with glutaraldehyde [97].

**Figure 15:**
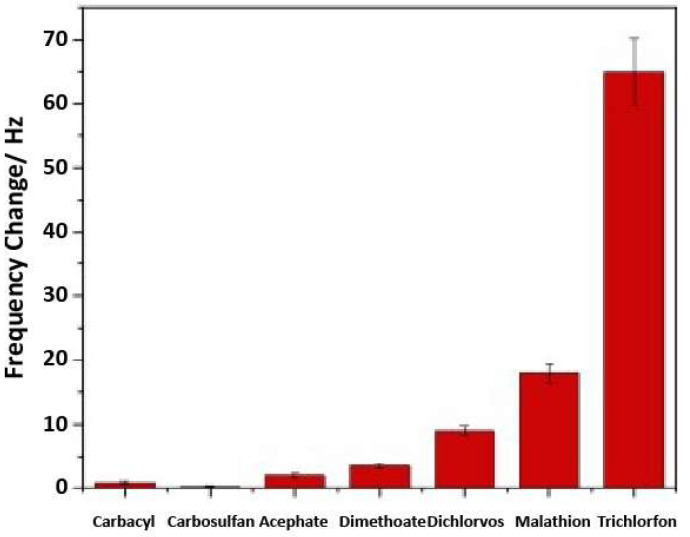
Selectivity of trichlorfon imprinted polymer at 100 ppb with various analogous compounds. MIPs were produced by combining 12 mg of polyvinylidene fluoride with 4 mg of template trichlorfon in 8 mL of dimethylformamide and drop-casting onto a quartz crystal. Reproduced with permission [111].

One final example of linear polymers was performed by Dayal et al., in their design of a QCM gas sensor for the organophosphate insecticide trichlorfon [111]. Poly(vinylidene difluoride) and trichlorfon (3:1 mass ratio) were dissolved in THF with poly(diallyl dimethylammonium chloride), which catalysed the formation of hydroxyl groups (via dehydrofluorination) on the polymer. The resulting MIP showed good selectivity when measured against several template structural analogues at 100 ppb, as shown in Figure 15. The MIP also showed a linear relationship with trichlorfon concentration, to approximately the 4.63 ppb limit of detection.

Pan et al., developed a SAW-based array sensitive to several organophosphate chemical weapons [113]. Targeting diisopropyl methyl phosphonate and dimethyl methylphosphonate (DMMP), sarin acid was used as the template. The results suggested a response proportional to concentration for both organophosphate targets with great sensitivity, in both cases below 1 mg m^−3^ (approximately 200 ppb) down to 0.05 mg m^−3^ (10 ppb). Hiller et al., similarly produced a dimethyl methylphosphonate sol-gel MIP sensor with an even lower reported detection limit of 13.3 ppt [18]. Additional sensors for weapons include the gas-phase detection of nitrotoluene explosives markers by Holthoff et al. [102]. The resulting instrument was sensitive to single ppb variations in dinitrotoluene concentration, giving a response linearly proportional to analyte concentration and a sub-ppb limit of detection.

### 4.3. Small Molecule Template Imprinting

Impressive selectivity and sensitivity can be achieved with gas sensing of larger targets, as previously shown. However, for many applications, and fair comparison with porogen imprinting, the focus will be given to smaller compounds. One of the most challenging targets of imprinted polymers is carbon dioxide. Amongst the most effective carbon dioxide adsorbents are those prepared by Chen and co-workers using pre-formed branched polyethyleneimine (PEI) [28,32]. The PEI polymer was dissolved in CO_2_ saturated water, after which glutaraldehyde was added as a crosslinker. The adsorption capacity was found to increase modestly with PEI molecular weight, but dramatically between imprinting and non-imprinting. Good selectivity for the target was found in the tests performed, with negligible adsorption of nitrogen under identical conditions. Low solubility of carbon dioxide can hinder this method however, and other researchers choose instead to use an analogue template [27,30]. Nabavi et al., conducted thorough studies of suitable reaction conditions for carbon dioxide adsorbent synthesis using oxalic acid as a template [29,30,31]. On studying the effect on CO_2_ capacity of variation in parameters (polymerisation time, temperature, concentration, etc.) the greatest determiner was found to be the presence of oxalic acid template in the polymerisation mixture [30]. This demonstrates the imprinting effect even for very small molecules with low template concentrations.

A similar method was used by Zhao et al., in their preparation of MIPs for nitrogen dioxide [35]. The group studied the effect of using acetic acid and ethanedioic acid as templates, using an acrylamide and ethylene glycol dimethacrylate as monomers. Ethanedioic acid was found to be most favourable for NO_2_ absorption, and the MIP was found to be largely unaffected by the presence of oxygen or water. Huang and Wang found that selective adsorbents for hydrogen sulfide (H_2_S) could be produced using water as a template [36]. This is particularly encouraging due to the dangers of working with the target H_2_S relative to those of the template analogue.

**Figure 16:**
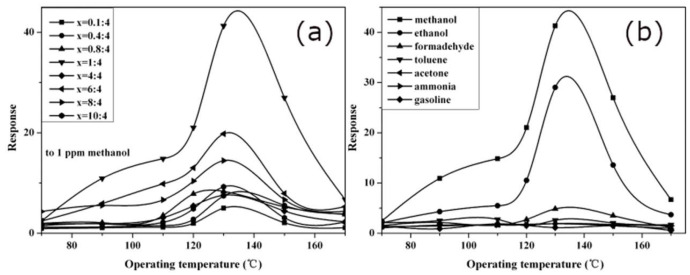
Methanol sensor developed by Zhu et al.: (**a**) effect of varying template-monomer ratio on response to 1 ppm methanol, (**b**) selectivity of 1:4 methanol-monomer sensor, showing response to 1 ppm of various gases. The functional monomer was methacrylic acid, and Ag-LaFeO_3_ was used as crosslinker (1:4:10 template-monomer-crosslinker). The resulting MIP was ground and printed onto an alumina electrode, and the response was recorded as the ratio of electrical resistance in the gas to that in air. Reproduced with permission [50].

Other examples of imprinting small molecules include a methacrylic acid and Ag-LaFeO_3_ material selective for methanol [50]. Zhu et al., tested ratios of template (methanol) and functional monomer (methacrylic acid) from 0.1:4 to 10:4, maintaining a 4:10 ratio functional monomer to crosslinking Ag-LaFeO_3_. The resulting ground monolith was printed onto an electrode for analysis, and the response for each ratio is shown in Figure 16a. The best response was found with a 1:4 template-monomer ratio, confirming that previous observations are applicable for imprinting small gas molecules. A study of ratio optimization in ammonia imprinting reached the same conclusion [54]. The selectivity of the methanol sensor was also good, displaying almost no response to many interferent molecules, though interference from ethanol was possible (Figure 16b). The sensor responded linearly in range of 1-5 ppm concentrations of methanol and gave a limit of detection of approximately 0.2 ppm. Follow-up work by the researchers showed a methanol gas sensor with linear response in the range 0.5–20 ppm, with a suppressed response to ethanol vapours [51].

### 4.4. Conclusions from Template Imprinting

Successful demonstrations of sensitivity and selectivity are generally easier to find in template imprinting, but this might be partially due to a bias in the data. Firstly, the greater number of examples of template imprinting give a greater opportunity for such demonstrations. Secondly, however, there is a lack of porogen imprinting with larger targets. This arises from a combination of sources, but results in fewer opportunities to work with targets likely to produce high affinity. With smaller targets, where template and porogen imprinting are both applied, the differences in affinity between the techniques are diminished. Selectivity however appears to be dominated by template imprinting regardless of the specifics of the target.

This difference is likely due to the previously discussed mechanisms. Template imprinting depends on a high ratio of polymer to template, giving a high proportion of polymer available for target binding. Porogen imprinting depends on probabilities of generating binding sites with a maximal number of template molecules. On average, this may under certain circumstances equate to an approximately equate detection limit between template and porogen imprinting. However, the precision of template imprinting will generally result in better selectivity.

Interference from humidity can still be a challenge for MIP gas sensors [76]. One unusual technique for overcoming this involved the use of pyrolysed lotus leaves in the polymerization mixture, which gave the final material greater hydrophobic properties [134]. This apparently arises primarily from the nanostructure of the leaf; similar increases in hydrophobicity have been observed by switching from MIP films to nanoparticles [21]. There could be an additional method of exploiting this however. As previously discussed, increases in imprinting factor, with sensitivity and selectivity, can be achieved by imprinting with oligomers or polymers followed by crosslinking [206]. It is possible therefore that biologically sources polymers could be used to improve MIP performance while simultaneously addressing environmental concerns. Recent examples of silk-based MIP nanoparticles for aqueous applications provide a foundation for this technology [207,208].

## 5. Conclusions

Porogen imprinting can result in degrees of selectivity and sensitivity which should be acceptable for many applications. Porogen imprinting may also be more suitable when working with certain materials, for example when attempting to create imprinted gas sensors or capture devices for common solvents. Conventional template imprinting however generally provides advantages over porogen imprinting in both sensitivity and selectivity. Template imprinting is therefore generally preferable for gas sensing applications. It is speculated that porogen imprinting and template imprinting may be used in tandem, for example in the development of a porogen imprinted switch. Such a material would therefore exhibit high affinity and sensitivity for a specific analyte in template imprinted binding sites only on exposure to a second porogen imprinted substance.

We have documented and categorized all the available examples of MIPs for gas phase analysis. From this it can be demonstrated that this is significant area of research, though small on the scale of molecule imprinting generally. MIP gas sensors provide high selectivity, a primary advantage over alternative gas sensor techniques. Multiple examples have also been given of MIP-based technologies generating responses to parts-per-billion or parts-per-trillion analyte concentrations. However, the standard of research overall is relatively low and the procedures quite basic in most cases, likely a result of more conservative approaches being taken in this less explored subject. Further advances in gas phase MIP applications are likely to emerge from greater deviation from standards set in liquid analysis and more thorough analysis of specific parameters. The efficacy of molecular imprinting in gas sensors has however been well established, and the quantity and quality of research in this area is only likely to increase.

## Figures and Tables

**Table 1:** Molecules targeted by gas phase molecular imprinting. Attempts were made to include a record of every example of gas phase applications of molecularly imprinted polymers in the literature. Targets are categorised by number of atoms and the imprinting technique used in the MIP synthesis.

Size	Template Imprinting	Porogen Imprinting	Structure
Small targets ≤ 10 atoms	Carbon dioxide [27,28,29,30,31,32,33], carbon monoxide [34], nitrogen dioxide [35], hydrogen sulfide [36], propenoic acid [37,38], ammonia [39], acetone [40,41], formaldehyde [16,21,42,43,44,45,46,47], furan [8], acetaldehyde [48], 1,3-dichloropropene [49], methanol [50,51], hydroxyl radical [52], ammonia [53,54]	Nitromethane [55], ethanol [56,57,58,59,60,61,62,63,64], methanol [22,60,65,66,67], formaldehyde [65,68], acetone [65,69], acetonitrile [41,70], water [61,71,72,73], chloroform [26]	Films: 42% Monoliths: 35% Nanoparticles: 24%
Medium targets 11-25 atoms	Hexanal [74,75,76,77,78,79,80], hexanoic acid [23,37,81,82,83,84,85,86,87,88], propanoic acid [23,81,85,86,88], hexanone [81], heptanone [81], heptanal [23,37,79,89,90], acetoin [91], phenol [91], multiple aromatics [92,93], benzaldehyde [77], trinitrotoluene [9,10,19,94,95,96,97,98,99,100,101,102], octanone [23,81,89], heptanoic acid [23,85,86,88,90], hydroquinone [103,104], toluene [105,106], benzaldehyde [90], 2,4-dinitrotoluene [11,97,107,108,109], 4-nitrotoluene [110], trichlorfon [111], nitrobenzene [112], dimethyl methylphosphonate [18,113,114], diisopropyl methyl phosphonate [113], pyridine [26], benzene [73,106], naphthalene [115], trimethylamine [53], 2-phenylethanol [116], (R)-α-methylbenzylamine [117], histidine [118], carvacrol [119]	3-Nitrotoluene [55], toluene [24,26,70,120,121,122,123,124,125,126], phenol [124], propionic acid [127], hexanoic acid [127], 4-ethylguaiacol [128], 4-ethylphenol [129], xylene [24,62,122,130,131,132,133,134,135,136,137], benzene [26,65,70], isopropanol [60], 1-butanol [61,71,72,73,138], ethyl acetate [44,62,71,72,73], 1-propanol [71,72,73], methyl benzoate [139], estragole [140], methyl salicylate [20], dimethyl methylphosphonate [141], tetrahydrofuran [62,64]	Films: 65% Monoliths: 20% Nanoparticles: 15%
Large targets >25 atoms	Geraniol [142], octanoic acid [23,37,38,81,85,86,88,89], limonene [143,144,145,146,147,148,149,150], limonene oxide [149], turmerone [151], curlone [151], ethyl-p-methoxycinnamate [151], cis-jasmone [147,152,153], linalool [150,154], geranial [155], neral [155], borneol [155], geraniol [150,155], nonanal [77,78,79,156], α-terpinyl acetate [157], α-pinene [146,147,149,150,158,159,160,161,162,163,164], β-phellandrene [158], 3-carene [158], cis-thujopsene [158], butylated hydroxytoluene [12,165], decanoic acid [166], nonanone [81], γ-terpinene [146,147,160,161], terpinolene [160,161], nicotine [167], adenosine monophosphate [14,168], pinacolyl methylphosphonate [169], parathion [170], 2-methylisoborneol [171], L-menthol [172], macromolecules [73,115,173,174,175,176]	Octanoic acid [127], heptane [61,177], limonene [71,72,73,140], α-pinene [140], β-pinene [140], eucalyptol [140], terpinene [140], diazinon [178]	Films: 88% Monoliths: 6% Nanoparticles: 5%
